# Do Ecological Niche Model Predictions Reflect the Adaptive Landscape of Species?: A Test Using *Myristica malabarica* Lam., an Endemic Tree in the Western Ghats, India

**DOI:** 10.1371/journal.pone.0082066

**Published:** 2013-11-29

**Authors:** Shivaprakash K. Nagaraju, Ravikanth Gudasalamani, Narayani Barve, Jaboury Ghazoul, Ganeshaiah Kotiganahalli Narayanagowda, Uma Shaanker Ramanan

**Affiliations:** 1 Department of Crop Physiology, University of Agricultural Sciences, Bangalore, Karnataka, India; 2 School of Ecology and Conservation, University of Agricultural Sciences, Bangalore, Karnataka, India; 3 Conservation Genetics Laboratory, Ashoka Trust for Research in Ecology and the Environment, Bangalore, Karnataka, India; 4 Department of Forestry and Environmental Sciences, University of Agricultural Sciences, Bangalore, Karnataka, India; 5 Ecosystem Management, Institute for Terrestrial Ecosystems, Zurich, Switzerland; 6 Biodiversity Institute, University of Kansas, Lawrence, Kansas, United States of America; 7 Department of Biology and Centre for Structural and Functional Genomics, Concordia University, Montréal, Québec, Canada; 8 Québec Centre for Biodiversity Science, Montréal, Québec, Canada; Institut Pluridisciplinaire Hubert Curien, France

## Abstract

Ecological niche models (ENM) have become a popular tool to define and predict the “ecological niche” of a species. An implicit assumption of the ENMs is that the predicted ecological niche of a species actually reflects the adaptive landscape of the species. Thus in sites predicted to be highly suitable, species would have maximum fitness compared to in sites predicted to be poorly suitable. As yet there are very few attempts to address this assumption. Here we evaluate this assumption. We used Bioclim (DIVA GIS version 7.3) and Maxent (version 3.3.2) to predict the habitat suitability of *Myristica malabarica* Lam., an economically important tree occurring in the Western Ghats, India. We located populations of the trees naturally occurring in different habitat suitability regimes (from highly suitable to poorly suitable) and evaluated them for their regeneration ability and genetic diversity. We also evaluated them for two plant functional traits, fluctuating asymmetry – an index of genetic homeostasis, and specific leaf weight – an index of primary productivity, often assumed to be good surrogates of fitness. We show a significant positive correlation between the predicted habitat quality and plant functional traits, regeneration index and genetic diversity of populations. Populations at sites predicted to be highly suitable had a higher regeneration and gene diversity compared to populations in sites predicted to be poor or unsuitable. Further, individuals in the highly suitable sites exhibited significantly less fluctuating asymmetry and significantly higher specific leaf weight compared to individuals in the poorly suitable habitats. These results for the first time provide an explicit test of the ENM with respect to the plant functional traits, regeneration ability and genetic diversity of populations along a habitat suitability gradient. We discuss the implication of these resultsfor designing viable species conservation and restoration programs.

## Introduction 

The concept of an ecological niche, proposed as early as 1900’s refers to a set of ecological conditions within which a species is able to maintain its population [1,2]. Grinnell [2] was the first to explore the relation between ecological niche and geographic distribution of species. He argued that ecological niche of a species is a key determinant that governs and limits the geographic distribution of species. An implicit assumption of the ecological niche is that, a species would maximize its fitness within its ecological niche than outside of it [3,4]. This assumption is based on the fact that over evolutionary time, species would be selected to adapt to a set of variables, characteristic of its ecological niche. Accordingly, it has been argued that, the fitness of a species would be highest at the foci of species’ ecological niche and would gradually decrease away from it [5]. Thus, viewed from the perspective of fitness, the ecological niche of a species should reflect the fitness or adaptive landscape of the species. However as yet there is no explicit validation of the above assumption. 

In the last two decades, a number of efforts have been made to formally treat the concept of ecological niche of a species using several modeling tools e.g. Bioclim[6],GARP [7], Maxent[8], that make it possible to identify specific sites where a species can thrive best. Essentially, the ENMs provide prediction on the range of habitats on a probabilistic scale, from least suitable to highly suitable, for a given species [9-12]. These correlative predictive models combine known geographic locations of a given species with underlying environmental data to identify suitable sites for given species and then map this information to predict the species geographic distribution. The ecological niche models have been used in a wide range of applications such as in locating rare and threatened species and habitats [13,14], rationalizing choice of habitat for species re-introduction [15], predicting the spread of invasive species [9], predicting the spread of crop pests [16] and in estimating the response of species to global climate change [17]. 

A number of studies have validated the predictions of the ecological niche models in identifying the distribution of the species. Most of these validations have been made using the presence/absence data of the species in those predicted habitats [18,19]. However, none of these studies have attempted to directly test the robustness of the niche predictions with respect to the species fitness in that habitat. In a recent study, attempts have been made to correlate the habitat suitability predictions with the natural species distribution and abundance; species abundances were negatively correlated with distance from the niche centroid of the species [20]. Keeping everything else constant, this suggests that species tend to be more abundant in sites predicted to be highly suitable compared to sites predicted to be poorly suitable. 

In this paper, we argue that the habitat suitability of a species as predicted by the ecological niche model may also reflect the adaptive landscape of the species. Thus population of species in sites predicted to be highly suitable would be expected to have a higher fitness than con-specific populations in sites predicted to be poorly suitable [4,5,20,21]. We examine this hypothesis empirically using an endemic and economically important tree *Myristica malabarica* Lam., in the Western Ghats, India. First, using the existing natural distribution data of the species, we generate ecological niche model predictions on the habitat suitability of *M. malabarica* in the Western Ghats. Second, we evaluate the fitness of the species in areas predicted to be highly suitable and poorly suitable using several direct and indirect measures of fitness [19,22-25]namely, a) plant functional traits - fluctuating asymmetry and specific leaf weight, b) recruitment or regeneration index and c) population genetic variability. We discuss these results in the larger context of ecological niche theory and models and their applications in conservation and management of economically important tree species.

## Materials and Methods

### Ethics statement

*Myristica malabarica* is an economically important plant, endemic to India. Field work was carried out in the central Western Ghats regions of Karnataka, with due permission from the Karnataka Forest Department. Tissue sampling was carried out under the supervision of local foresters and used solely for scientific research. The sampling was non-invasive and does not in any way affect the natural growth of *M. malabarica*.

### Study site

The study was conducted in the Western Ghats, a mountain chain running parallel to the West coast of India and one of the 34 biodiversity hotspots of the world [26]. Occupying about 5 % of India’s landmass, it harbors about 27% of the country’s plant species, with more than 60% endemic to the region [26]. The Western Ghats has over 4500 flowering species including economically important trees from Dipterocarpaceae, Myristicaceae and other families [27]. The climate ranges from tropical wet dry to tropical wet with elevation ranging from 1500 metres AMSL in the north to 2000 metres AMSL in the south. Mean temperature varies from 24 °C in the north to 20 °C in the south. The Western Ghats receive an average of 3000 to 4000 mm rainfall with occasional rainfall of 9000 mm. The present study was conducted in central part of Western Ghats between 12° - 14° north and 74° - 75° east ( Table S1 in File S1). The study area has a tropical climate with a well-defined rainy season; between June and November the region receives rainfall ranging from 300 to 2500 mm with temperature ranging from 15 °C - 25 °C. The study was conducted during 2008 - 2010.

### Study system

*Myristica malabarica* Lam. commonly known as “Bombay mace, Malabar Nutmeg or Jaikai” belongs to the family Myristicaceae[28]. It is an evergreen tree, growing up to 15-20 m in height. The species is distributed in the Western Ghats, occasionally along freshwater streams and most frequently in evergreen and semi-evergreen forests [28-30]. The trees bear arillated fruit which is harvested and used as a condiment. In recent years, because of extensive and indiscriminate harvest of its fruits, the regeneration of the species is severely affected. Furthermore, most of the natural habitat of *M. malabarica* has also been fragmented due to human activities leading to decline in the natural populations of *M. malabarica*. Owing to these pressures, the species has been designated as ‘vulnerable’ [29]. Considering the threat and economic importance of *M. malabarica*, in recent years there is a growing interest in domesticating the species and in prioritizing areas for its conservation. 

### Ecological niche modeling

Based on a number of field surveys undertaken over a two year period (2008 to 2010) in the Western Ghats, we recorded the latitude and longitude of 56 sites of occurrence of *M. malabarica* using a global positioning system (GPS-Garmin12). We used this primary presence only data for modeling the species distribution [31]. 

We used three algorithms[32]to develop the potential distribution and generating the suitability surfaces for the species, namely, a) Bioclim (Bioclimatic analysis and prediction system,) - this is based on presence only data and was run using the envelope method using DIVA-GIS software, b) GARP (Genetic Algorithm for Rule Set Prediction) - this uses pseudo-absences to fit logistic regression to the data using Open Modelers Best subset implementation protocol, c) Maxent, (Maximum entropy) - this uses background points and minimizes the entropy. The softwares used were MaxEnt (version 3.3.2), DIVA-GIS (version 7.1.7.2; http://www.diva-gis.org) and GARP (version 1.1.3). We used 19 bioclimatic variables generated by Hijmans et al [33]; these were downloaded from http://www.worldclim.org/ with 30 seconds (~1km) spatial resolution. These variables represent combinations of temperature and precipitation, which are fundamental to species survival. We also built a model with elevation and 19 bioclimatic variables, but the model performance was not better than random. Though additional layers such as disturbance, invasive species presence, forest type, and other possible variables could have been used, these may often overfit the model [34]. Also ecology of the species suggests that temperature and precipitation variables are sufficient to estimate its presence [30] and our field observation suggests that there were no substantial differences among the sites with respect to the other variables. Species occurrence data was obtained from both literature and field survey. We divided the 56 occurrence points into 2 sets of 50% each, one set for calibration of model and the other set for evaluation of model performance. We applied a buffer of 100 km around Western Ghats to generate the calibration area for the model. Barve et al [35], discusses the effect of calibration area in training the model. We used the default setting for all the 3 models while training. 

We used Least Training Presence Thresholding method for converting the prediction into habitat suitable indices [36]. In this method, we assigned the probability of presence to each occurrence point and considered lowest suitability score as the least score where species could be present. Bioclim and Maxent uses continuous probability scale while GARP uses integer scale; we multiplied Bioclim and Maxent prediction by 100 to have comparable scores among these models. 

We used these calibrated models to evaluate the model performance using the set of occurrence points that was not used in building the model. As the classic area under curve (AUC)-Receiver operating curve (ROC) gives equal weight to commission and omission error [37], it is not greatly applicable to the modeling algorithm where presence only data is used for training the model. We used partial ROC method for testing performance of the model against null expectation of random model. The model calibration was better than random with p-value < 0.05. 

**Figure 1:**
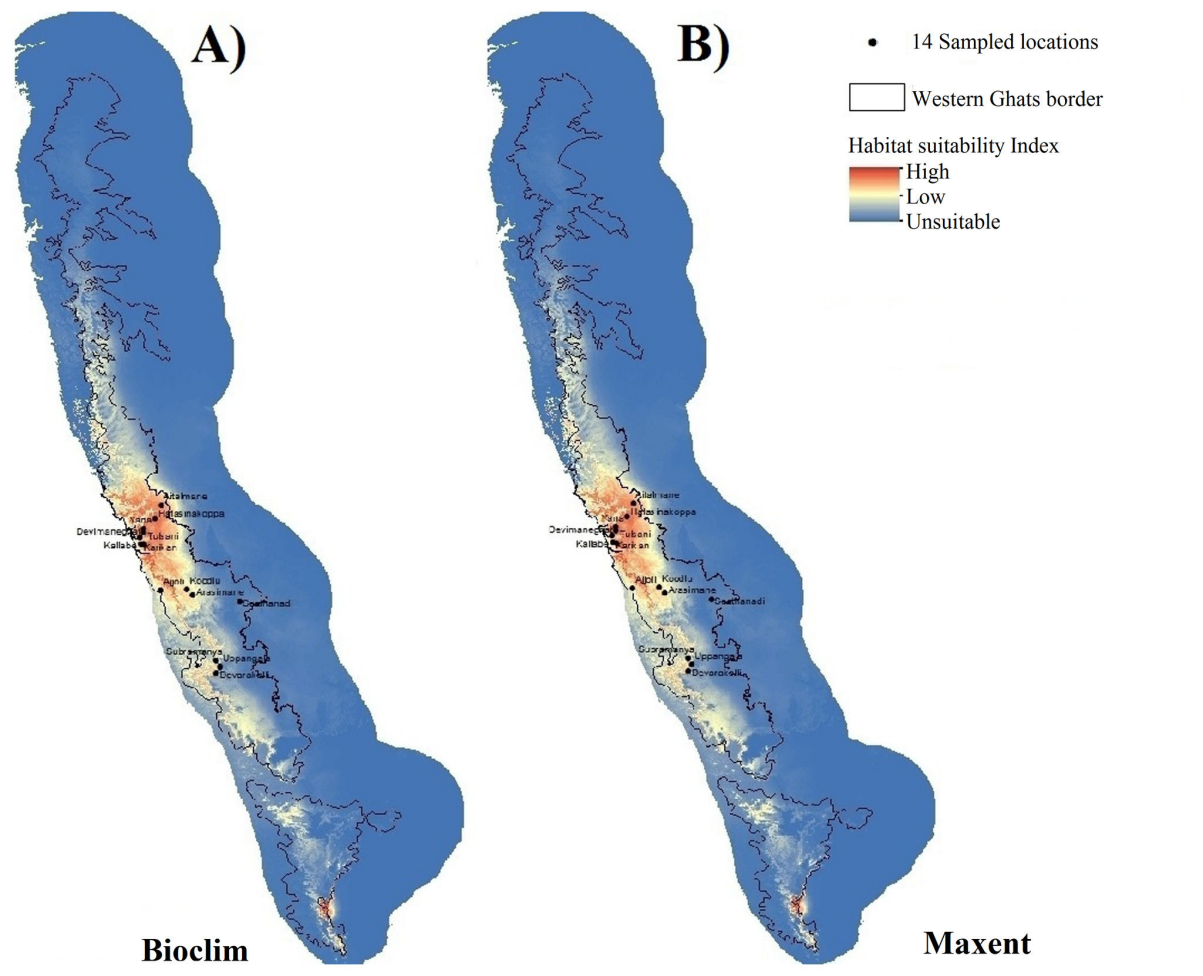
Ecological niche prediction map of *Myristica malabarica* in the Western Ghats, India. A) Bioclim B0 Maxent.The sites at which the plants were sampled is also shown. .

Of the three models, GARP did not sufficiently discriminate sites based on their habitat suitability values, and hence we did not consider the model prediction further. On the other hand, Bioclim and Maxent discriminated the sites based on their habitat suitability; furthermore the discrimination was comparable between the models (see Figure 1 and Table S1 in File S1). For all further purposes of the study, we therefore restricted our analysis to Bioclim and Maxent models only. 

Based on the model predictions, we selected sites representing different habitat suitability indices to sample individuals and populations to evaluate their fitness. For the purpose of analysis, we considered the 14 sites as a continuum of habitat suitability indices. The sites are separated geographically ranging from 10 to 400 km in the Western Ghats (Table S1 in File S1). 

### Predictions

Specifically we ask if populations of the species in sites predicted to be highly suitable (with higher habitat suitability index) have a higher fitness than con-specific populations in sites predicted to be poorly suitable (with low habitat suitability index). Traditionally, the unambiguous measure of fitness is fecundity or reproductive output [38-41]. However, in canopy trees, such as *M. malabarica* this is rarely feasible to be measured. For the purpose of this study, we chose three measures of species performance that have in the past been used as measure of fitness, namely: a) plant functional traits - fluctuating asymmetry (FA) and specific leaf weight (SLW), b) recruitment or regeneration index and c) population genetic variability. Accordingly, we make the following specific predictions:

#### Prediction 1

*Individuals in areas predicted to be highly suitable (by the ecological niche models*)* would be expected to have a low FA (and hence higher fitness*)* in contrast to sites predicted to be poorly suitable*. 

#### Prediction 2

*Individuals in areas predicted to be highly suitable (by the ecological niche models*)* would be expected to have a higher SLW (and hence contribute to higher fitness*)* in contrast to sites predicted to be poorly suitable*. 

#### Prediction 3

*Populations in sites predicted to be highly suitable (by the ecological niche models*)* would be expected to have a higher regeneration index compared to those sites predicted to be poorly suitable.*

#### Prediction 4

*Populations in sites predicted to be highly suitable (by the ecological niche models*)* would be expected to have a higher genetic diversity compared to those sites predicted to be poorly suitable.*

### Test of predictions

At each of the 14 selected sites of varying habitat suitability, the data on the following were collected. The geographic range of *M*. malabarica and 14 sampling locations is given in Figure S1.

### Fluctuating asymmetry and specific leaf weight

Fluctuating asymmetry (FA) is a widely used measure of developmental instability [42,43]. An individual unable to buffer random accidents of development, whether genetic or environmental in origin, may exhibit deviation from perfect symmetry [44]. Such deviations which are non-directional and random are termed as fluctuating asymmetry. Although FA has traditionally been used to measure developmental instability of populations [45], this concept has been extended to address various other ecological questions. According to FA model, less asymmetric (more symmetric) individuals have greater developmental stability, better survival rate, greater reproductive success and fitness. In this study, we used FA as a surrogate of fitness (or more precisely lack of fitness).

Matured leaves from 10 trees (branches were randomly chosen for sampling) were collected from each of the 14 sites. For each leaf we measured width of the right and left halves from the midrib to the leaf margin at the mid-point (half-way between the base and tip), perpendicular to the midrib. Leaf fluctuating asymmetry was then calculated as: FA = (L – R)/size, where L =width of the left side, R = width of the right side, and size = (L + R)/2 [46].

Specific leaf weight (SLW) is the ratio of leaf mass to leaf area. Physiologically, SLW refers to the density of packing of chloroplast; a higher SLW would indicate a higher photosynthetic efficiency and by extension can be expected to be positively related to fitness of the individual. 

The specific leaf weight was measured as a ratio of leaf dry mass (mg) to its area (mm^2^). Ten matured leaves from each of 10 trees were harvested from each of the 14 populations. Leaf discs (1 cm diameter) were punched and oven dried at 70 °C for 72 h before being weighed. SLW was calculated as follows

$$SLW\text{= dry weight of the leaf disc }\left(\text{mg}\right)\text{ /area of the leaf disc }\left({\text{mm}}^{\text{2}}\right)$$

### Regeneration index

Regeneration index is a direct reflection of the life history strategy of a plant. It integrates the sum total of events from reproduction to the successful establishment of progeny and thus can be regarded as a measure of “realizable” fitness [47,48]. At each of the 14 sites, 20 quadrats (10*10) were laid and data on density of adults per quadrat and girth at breast height (GBH)of all individuals in a quadrat was recorded [49]. The regeneration per quadrat was assessed by recording the number of seedlings and saplings (< 1cm GBH) in 1 m^2^ nested quadrats at two diagonal ends of each of the 20 quadrats. As an index of regeneration per adult, we computed the ratio of number of seedlings and saplings (<1cm GBH) to the total number of adults per quadrat.

### Genetic diversity

Population genetic variability at neutral markers such as simple sequence repeats (SSR) indicates the sum of mating events in a population and hence integrates a wide variety of parameters including population density, gene flow, recruitment success etc. This measure has been used in the past as a measure of population fitness. For example, several earlier studies have reported a direct association between the population genetic variability and fecundity in plants and animals [50-52]. 

Population genetic diversity estimates were determined for 12 of the 14 sites (for two sites, samples could not be genotyped). Fresh leaf samples were collected from 10-25 individuals per site. Since the species is a canopy tree, we resorted the services of tree climbers to access the tree crown and to fetch the desired leaf sample. The leaves were dried in silica gel and stored at -40 °C until DNA extraction. Total genomic DNA was extracted from the leaves following a standard CTAB procedure [53]. DNA quantification was performed by comparison with known concentration of a DNA standard (Lambda DNA) in ethidium-bromide stained 1% agarose gel.

Microsatellite marker analysis of the populations (n=194 individuals) representing the 12 sites was carried out using 5 polymorphic primer pairs out of 11 published SSR primer pairs developed for *M. malabarica* [54]. The protocol for microsatellite DNA marker analysis at the 5 chosen loci is described in Hemmilia et al [54]. 

The 5’ end of forward SSR primers were labelled with fluorescent dye (FAM1, FAM2, NED Y, VIG G and PET R) and samples for genotyping were prepared by mixing 10 μL of deionized formamide, 0.1 μL of 35-500 bp internal size standard (Applied Biosystems, Chromos Biotech) and 1 μL of PCR product. The mixture was denatured at 95 °C for 2 min and immediately placed on ice for a minimum of 5 minutes and loaded onto an ABI 310 Genetic Analyzer (Applied Biosystems, Chromos Biotech) for capillary electrophoresis and fluorescent scanning detection. The electropherograms of genotypes were analyzed using GeneScan 3.7 and Genotyper 3.7 (Applied Biosystems).

The data was subjected to population genetic analysis to measure the overall genetic variability of populations sampled across the different habitat suitability regimes predicted by ENM. The following measures were calculated using different population genetic software: the mean number of alleles per locus (averaged across 5 loci) and to avoid bias caused by uneven sampling, a standardized estimate of allelic richness and private alleles (alleles that are exclusive to a population and habitat) independent of sample size [55] was calculated using the program FSTAT 2.9.3 [56] and HP-RARE [57]respectively.The gene diversity per locus (averaged over 5 loci) was calculated using program FSTAT 2.9.3[56]. Test for linkage-disequilibrium and Wright’s F-statistics (Fis and Fst) was assessed using GENEPOP 3.2a [58].

### Statistical analysis

We used general linear model (GLM) to relate the three fitness measures (functional traits, regeneration index and genetic diversity parameters) with habitat suitability index. The three fitness measures were defined as the response variables and the habitat suitability index as a fixed explanatory variable. The relationship of the three fitness measures, namely, functional traits (FA and SLW), regeneration index and genetic diversity parameters with habitat suitability index was also evaluated using simple linear regression models. Besides, we also analysed for differences in the frequency distribution of FA and SLW across highly and poorly suitable habitats using a non-parametric Kolmogorov-Smirnov (KS) two sample test[59]. For the Bioclim model we categorized the sites into highly suitable (all sites with habitat suitability index > 100) and poorly suitable (all sites with habitat suitability index < 50). Similarly, for the Maxent model we categorized the sites into highly suitable (all sites with habitat suitability index >70 and poorly suitable (all sites with habitat suitability index <45). The deviation of frequency distribution for FA and SLW from normality was tested using skewness test. To minimize sampling bias, data on both FA and SLW were randomized 25 times and at each time 50% of randomized data was subjected to two sample KS and skewness test for the frequency distribution of FA and SLW across highly suitable and poor habitats (Table S2 and S3 in File S1).

As a possible alternative explanation, we also analyzed if the population genetic parameters were correlated with the latitude from where the samples were collected. The relationship was statistically evaluated using univariate general linear models [60]. 

## Results

### Calibration of models and niche model analyses

The model calibration test using partial ROC statistics for *M. malabarica* yielded satisfactory results (Bioclim: ROC p<0.001, MAXENT: ROC p<0.0001). Amongst the 19 input Bioclim variables only 12 contributed to model variation (Table 1). Among these 12 variables, annual precipitation (bio2) contributed more to the model (44.5%) followed by mean temperature of wettest quarter (bio18) (13.3%) and precipitation of driest quarter (bio17) (12%). All three variables together contributed 69.8% of variation to the model (Table 1). The remaining 9 variables together contributed 30.3% variation to the model (Table 1). Considering the permutation importance, only 11 out of 19 variables contributed to the model variation. Among the 11 variables, temperature seasonality (bio4) (36%) and precipitation of wettest month (bio13) (34.7%) had maximum influence on habitat suitability model followed by mean temperature of wettest quarter (bio8) (12.2%). All three variables together contributed to 82.9% of the variation; the remaining 8 variables together contributed to 12.9% (Table 1).

**Table 1:** Estimates of relative contribution and permutation importance of 19 bioclim variables to Maxent model.

**Variables**	**Percent contribution**	**Permutation importance**
Annual precipitation (bio12)	44.5	0.1
Mean temperature of wettest quarter (bio8)	13.3	12.2
Precipitation of driest quarter (bio17)	12	4.9
Precipitation of coldest quarter (bio19)	9.6	0.2
Precipitation of wettest month (bio13)	6.8	34.7
Mean temperature of coldest quarter (bio11)	5.2	5.3
Precipitation seasonality (bio15)	3.5	2.9
Precipitation of warmest quarter (bio18)	2.7	2.7
Isothermality (bio3)	1.7	0.9
Temperature seasonality (bio4)	0.4	36
Maximum temperature of coldest month (bio6)	0.3	0
Annual mean temperature (bio1)	0.1	0
Mean temperature of driest quarter (bio9)	0	0.1
Precipitation of driest month (bio14)	0	0
Maximum temperature of warmest month (bio5)	0	0
Temperature annual range (bio7)	0	0
Mean monthly temperature (bio2)	0	0
Mean temperature of warmest quarter (bio10)	0	0
Precipitation of wettest quarter (bio16)	0	0

Both models, Bioclim and Maxent, predicted the current distribution of *M. malabarica* that was concordant with the known distribution range of the species in the Western Ghats (Figure 1). However as seen from the figure, not all areas in the Western Ghats region are uniformly suitable. There are distinct patches in the Western Ghats that are predicted to be very highly suitable (dark red regions) as opposed to certain patches that are poorly suitable (light yellow) or not suitable (blue) at all. Most of the highly suitable sites are located in the Central Western Ghats. The two models sufficiently discriminated the 14 sites selected for the study based on their habitat suitability indices (Figure 1 and Table S1 in File S1). 

### Fluctuating asymmetry and specific leaf weight

**Figure 2:**
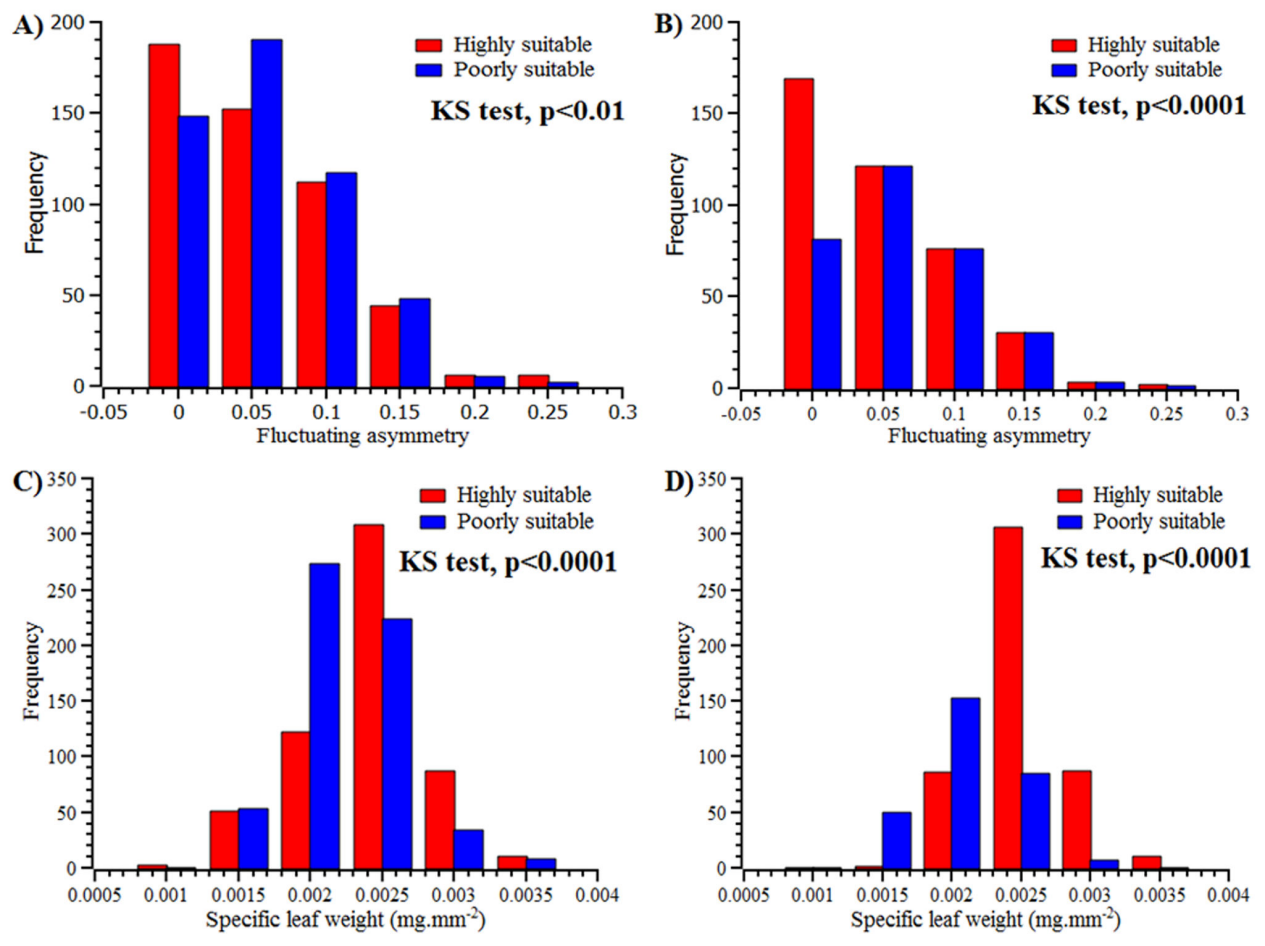
Frequency distribution of fluctuating asymmetry (A: Bioclim; B: Maxent) and specific leaf weight (C: Bioclim; D: Maxent) for highly suitable (blue) and poorly suitable (red) habitats (for details see text).

Fluctuating asymmetry of leaves differed significantly among the sites (Bioclim= F=1.96, df=7, p=0.057; Maxent = F=3.22, df =10, p<0.0001) (Table 2). Leaves of individuals in sites predicted to be highly suitable were less asymmetric compared to those in poorly suitable habitats; for nearly 45% of all individuals in the highly suitable habitat, the FA were very small indicating that they were indeed symmetric (Figure 2A and B). The frequency distribution of FA was sharply positively skewed for individuals from highly suitable habitat compared to those from poorly suitable habitat (Table S2 in File S1and Figure 2A and B).The frequency distribution of FA across highly suitable and poorly suitable habitat was significantly different (two sample KS test for Bioclim p<0.01, for Maxent p<0.0001; Table S3 in File S1). There was a significant relationship between habitat suitability index and FA under Maxent model (p=0.032) but not under Bioclim (p=0.268; Table 3). 

**Table 2:** Relationship between plant fitness traits and habitat suitability index under Bioclim and Maxent models. Results are based on univariate ANOVA under general linear model (GLM).

	**Models**				
**Fitness traits**	**Maxent**			**Bioclim**	
	**F(df)**	***P***		**F(df)**	***P***
Regeneration/adult	6.05(10)	<0.0001	8.61(7)		<0.0001
Specific Leaf Weight	83.94(10)	<0.0001		34.01(7)	<0.0001
Fluctuating Asymmetry	3.22(10)	<0.0001		1.96(7)	0.057
Gene diversity per locus	0.47(10)	0.903		0.80(7)	0.591
Observed number of alleles	7.56(10)	0.008		8.56(7)	0.005
Allelic richness	3.05(10)	0.08		3.29(7)	0.074
Number of private alleles	7.52(10)	0.008		9.48 (7)	0.0032

**Table 3:** Simple linear regression between habitat suitability index and plant fitness traits.

	**Models**			
**Fitness traits**	**Bioclim**		**Maxent**	
	**r-value**	**p-value**	**r-value**	**p-value**
Density	0.107	0.715	0.241	0.400
Regeneration/adult	0.705	0.01	0.719	0.008
Specific Leaf Weight	0.111	<0.0001	0.346	<0.0001
Fluctuating Asymmetry	0.031	0.268	0.06	0.032
Gene diversity/locus	0.592	0.042	0.646	0.023
Number of private alleles	0.375	0.0032	0.339	0.008
Observed number. of alleles per locus	0.359	0.005	0.339	0.008
Allelic richness	0.232	0.074	0.224	0.086

**Figure 3:**
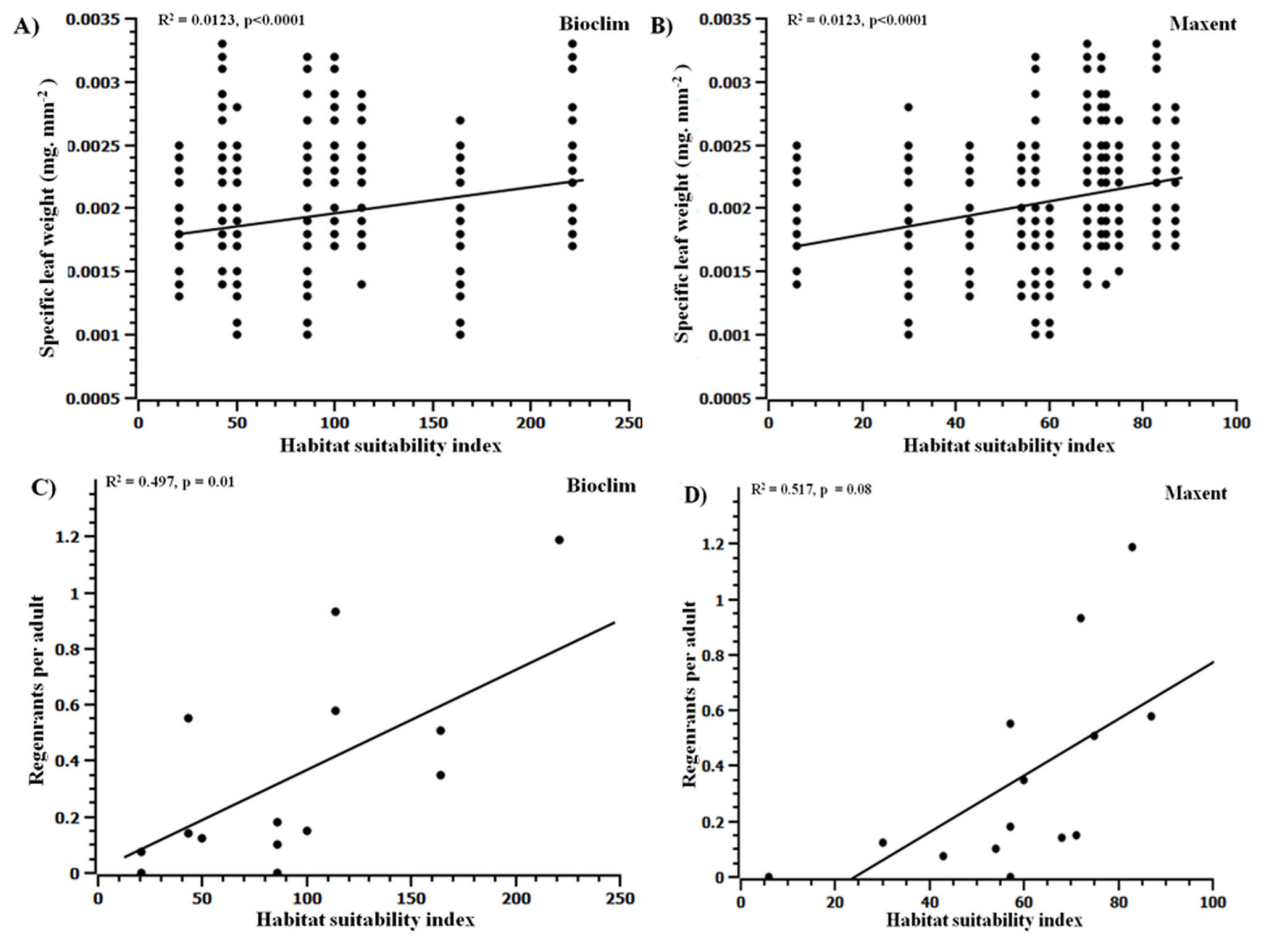
Specific leaf weight (A: Bioclim; B: Maxent) and regeneration index (C: Bioclim; D: Maxent) in relation to habitat suitability index.

Individuals occurring in highly suitable habitats had significantly higher SLW compared to those occurring in the poorly suitable habitats. The SLW was positively correlated with habitat suitability index (Figure 3A and B and Table 3). The frequency distribution of SLW was significantly different across the highly suitable and poorly suitable habitats (Table S3 and Table S4 in File S1, and Figure 2C and D). Finally, the univariate ANOVA under GLM showed that the SLW was significantly different among the different habitat suitability categories under both Bioclim and Maxent models (Table 2). 

### Regeneration Index

There was a significant positive relationship between the number of regenerants per adult (regeneration index) and habitat suitability index based on both, Bioclim and Maxent models (Figure 3 C and D and Table 3). Habitats that were predicted to be highly suitable had on an average significantly greater regeneration index compared to sites that were predicted to be poorly suitable. For example, the site, Seethanadi, that was predicted by both the models to be the least suitable (habitat suitability index= 21 under Bioclim and 6 under Maxent) had the least regeneration index (=0.2) compared to the site, Halasinakoppa (habitat suitability index of 221 (Bioclim) and 83 (Maxent) which had the highest regeneration index of 1.2. Univariate ANOVA under GLM also indicated that the regeneration index differed significantly among the predicted habitat suitability categories under both Bioclim and Maxent models (Table 2). The density of adults was not significantly correlated with the habitat suitability index (p= 0.107 under Bioclim and p=0.40 under Maxent). The spatial map of density of adults is given in Figure S2. 

### Genetic diversity

**Figure 4:**
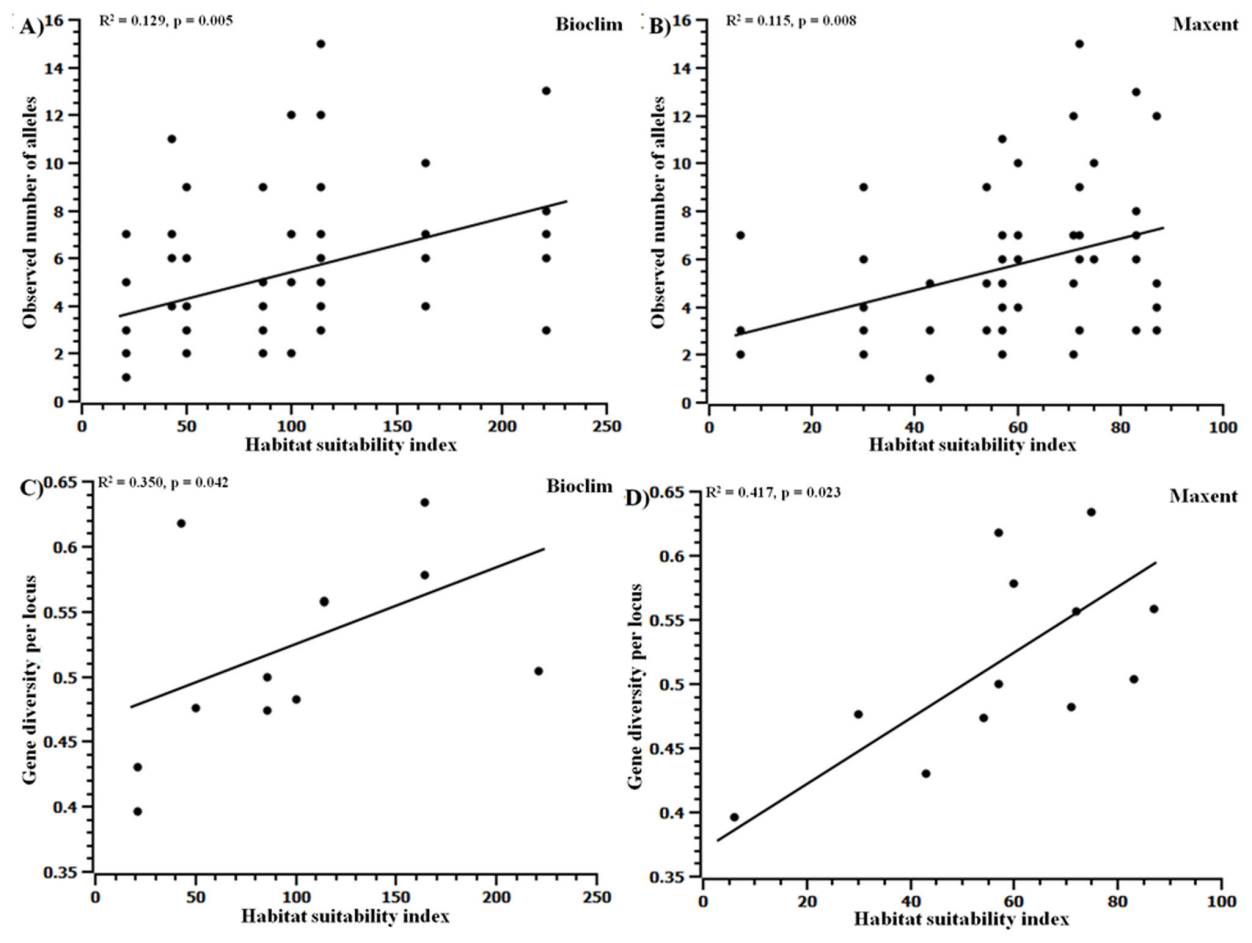
Population genetic parameters in relation to habitat suitability index. Observed number of alleles (A: Bioclim; B: Maxent) and Gene diversity per locus (C: Bioclim; D: Maxent) .

**Figure 5:**
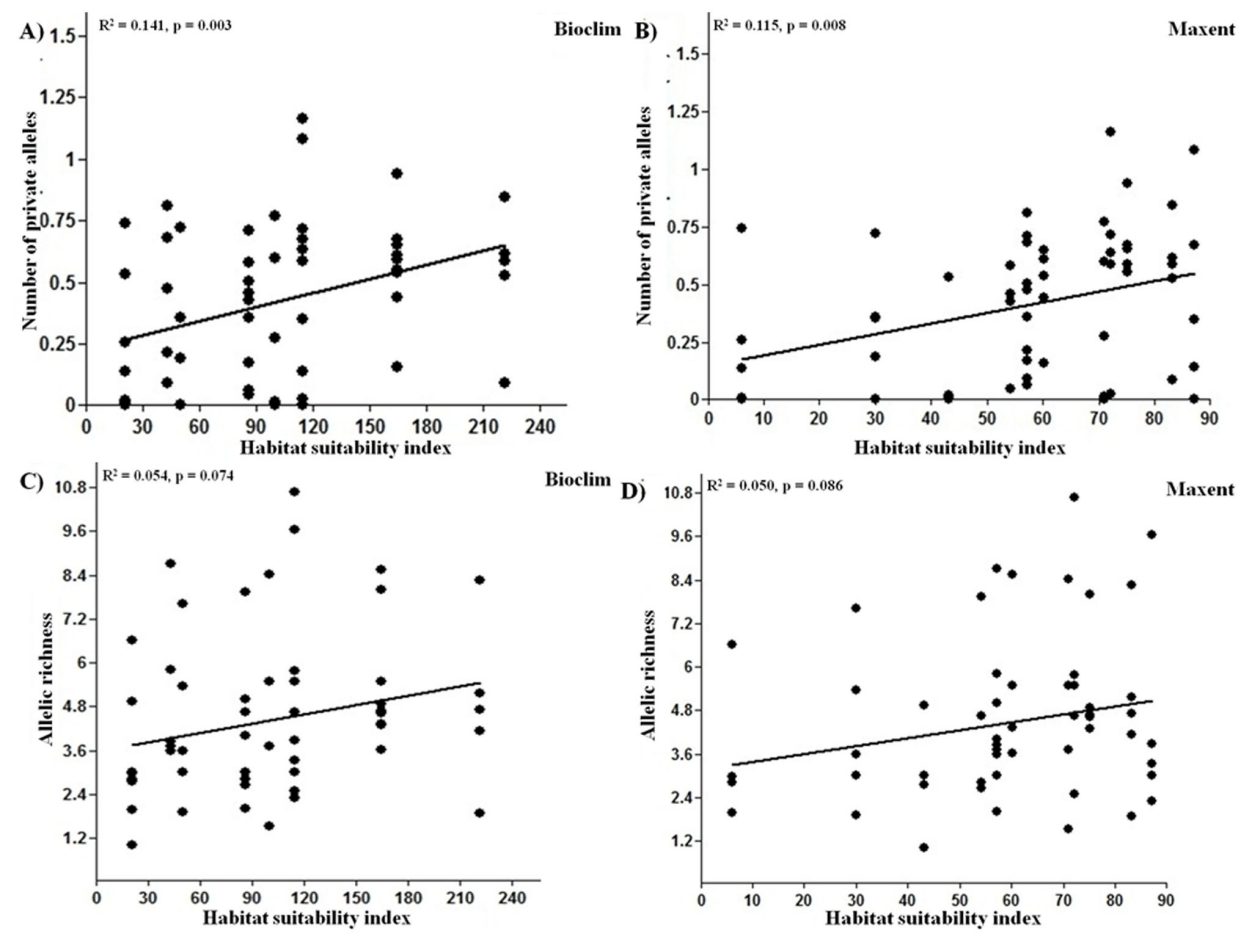
Population genetic parameters in relation to habitat suitability index Number of private alleles (A: Bioclim; B: Maxent) and Allelic richness (C: Bioclim; D: Maxent).

Over the 5 SSR loci, a total of 102 alleles were recovered from 194 individuals sampled from 12 populations. The mean allele number over all the loci for all the population was 15.4. Observed allele number per locus was significantly correlated with habitat quality (p=0.005 for Bioclim and p=0.008 for Maxent) (Figure 4A and B and Table 3). The, gene diversity per locus was also positively correlated with habitat quality under both Bioclim (p=0.042) and Maxent models (p=0.023) (Figure 4C and D and Table 3). The number of private alleles (defined as those alleles that are exclusive to the specific population) increased with predicted habitat quality (p = 0.0032 for Bioclim and p = 0.008 for Maxent) (Figure 5A and B and Table 3); for example there was a nearly five-fold increase in the number of private alleles from sites that was predicted to be least suitable (Seethanadi and Ajjoli) to sites that was highly suitable (Devimane, Tulsani and Halasinakoppa). The allelic richness also increased with predicted habitat suitability (p = 0.074 for Bioclim and p = 0.086 for Maxent) (Figure 5A and B and Table 3). There was no significant differences in the percent observed heterozygosity of the populations across predicted habitat suitability. The spatial map of allelic richness and genetic diversity are given in Figure S3.

Analysis of univariate ANOVA under general linear model (GLM) (Table 2) also indicated that population genetic parameters were significantly different among the sites (Table 2). However, gene diversity per locus was not significantly different across sites. Except two parameters, namely, observed number of alleles and pair-wise Fst, none of the genetic diversity parameters (allelic richness, number of private alleles and gene diversity per locus) were significantly correlated with latitude (Table S5 in File S1). 

## Discussion

In recent years a number of ecological niche modeling (ENM) tools have been used in predicting the habitat suitability of species [9,11,13]. An implicit assumption of ecological niche is that individuals of a species would maintain a higher level of fitness within its niche than outside. However, few studies have actually addressed the issue and fewer still have asked if indeed the predictions made by ecological niche models on habitat suitability reflect the variation in plant fitness and abundance [61–63]. Among various constraints, impeding such studies is the difficulty of accurately obtaining measures of plant fitness along a gradient of habitat quality [63]. In absence of robust measures of plant fitness, Violle et al. [22] have suggested that several functional traits (physiological, morphological or phenological) could be considered as surrogates of plant fitness as these often relate to the final reproductive output of the plants. 

In this study we considered three parameters of species performance, namely, plant functional traits (FA and SLW), regeneration index and genetic diversity. All measures are inter-dependent and reflect the cumulative effects of the life-history processes of a population, and have been used in the past to indicate the health of species’ population. For example, seed production or fecundity of plants (as a direct measure of fitness) has been found to be significantly higher in genetically diverse compared to inbred populations [64–66]; hence measures of genetic diversity have often been used as surrogates of fitness. As mentioned elsewhere, in our study, all the three fitness measures, the functional traits (FA and SLW), regeneration index and genetic diversity were significantly related to the predicted habitat quality. All these relationships are consistent with the predictions made by both the models, Bioclim and Maxent. 

Fluctuating asymmetry is a widely used measure of developmental instability. The instability could be genetically driven (as in developmental lethals) or environmental induced (as under stressful environments). Our study showed that individuals occurring in highly suitable habitats were significantly less asymmetric in their leaf morphology compared to those in poorly suitable habitat. In other words, it appears that individuals and hence population in highly suitable habitats might be more “fit” than those from the poorly suitable habitat. The difference in FA between the habitats might reflect the underlying genetic adaptations of respective populations; individuals in the highly suitable habitat being better adapted to their habitats than are individuals in the poorly suitable habitats. In the latter scenario, individuals may also been seen to be “stressed” in the poorly suitable habitats. Indeed, several studies have reported a positive association between leaf FA and environmental stress [67–69]. For example, leaf FA has been shown to increase with pollution loads [70], higher elevation [67] and colder climate [68]. Nagamitsu et al. [69] showed that drying of soil and competing with invading plants can also cause leaf FA.

Another functional trait, the specific leaf weight (SLW), is an indicator of leaf thickness and the degree of mesophyll development within a leaf blade. The extent of mesophyll development largely determines the photosynthetic capacity of a leaf, although intracellular effect or other factors such as nutrient supply may also influence photosynthetic capacity [71]. Clearly a higher SLW can be expected to contribute to the net fitness of a plant through enhanced resource supply for seed production. A significantly higher SLW of plants in the highly suitable habitat compared to plants in the poorly suitable habitats once again indicate the relative adaptive landscape of the plants. While in the former, photosynthetic process might be expected to have been optimized, clearly in the latter (poorly suitable habitats), these processes could be far from ideal. Besides, contributing to the net photosynthates, SLW can also contribute to deterring herbivore damage, and hence contributing to fitness gains [72,73]. 

The number of regenerants per adult was significantly higher for populations in highly suitable sites than in poorly suitable sites indicating that as a direct measure of fitness, it pays to be in habitats that are highly suitable. The higher number of regenerants per adult (seedlings and saplings) might arise due to a number of features in the populations in the highly suitable sites. These might range from a higher mating probability to a better survivability of the seeds in such habitats. It would be interesting to tease out these different variables in order to understand the critical role they play in shaping the lifetime fitness of plants across the gradient of habitat suitability. However, we did not find any significant differences in the density of adults across the gradient of habitat suitability. In other words, the increased regeneration in high habitat suitability sites does not seem to be translated in to increased density of adults. Lack of realization of recruits in to adults could be influenced by number of contemporary variables such as human disturbances, grazing pressures, man-made fire etc., all of which were observed at one time or the other during the field visits (personal observation). These variables are extraneous and do not reflect the intrinsic habitat quality. Thus the differences in recruitment between the highly and poorly suitable habitats may actually reflect the real population differences due to differences in the habitat suitability.

Our study also demonstrated that observed mean number of alleles per locus (N_A_) and gene diversity per locus were significantly affected by habitat suitability. Also the highly suitable habitats had higher number of private alleles compared to poorly suitable habitats. These differences across the habitat suitability could arise due to evolutionary ecological processes. The highly suitable habitat may in fact indicate the geographical foci of the species origin and diversification. Accordingly these sites would be expected to represent the allelic diversity of the species over the evolutionary time frame. On the other hand, the poor habitat might represent the limits of the species distribution and therefore could be expected to have shed a number of alleles due to genetic drift [74].

To further reiterate that the observed patterns, both in the demography and genetics, are a reflection of niche suitability, we also analyzed if these patterns were related to the latitudinal variations. Except two parameters, namely, observed number of alleles and Fst, none of other genetic diversity parameters were correlated with latitudinal variations, indicating that the overriding influence is indeed the habitat suitability. In summary, this is perhaps the first time that an explicit demonstration of niche model outputs has been empirically demonstrated to influence species fitness.

Recently, Dixon et al. [75]evaluated the “abundant center hypothesis” with respect to demographic and genetic parameters of *Leavenworthiastylosa*. They showed that demographic parameters (plant density, average seed number per plant) increased and genetic parameters (heterozygosity and allelic richness) decreased with increasing distance from the “abundant center” of species distribution. Assuming that the abundant center of the species may actually represent the niche centroid as proposed by Meyer et al. [21] it is clear that the genetic diversity of the species is shaped by the habitat suitability as is evident from our study.

But why should predictions about habitat suitability reflect variation in plant fitness? Albert and Thuiller [76] argued that to the extent that ecological niche models actually depend on and base their prediction on the frequency occupation of habitats by a species, it is likely that “among habitats occupied by the species, the more frequent (habitats) are also the more suitable”. In a categorical analysis of species records in different habitat suitability types predicted by Bioclim and Maxent, we found that there was a greater than expected occurrence of species in habitats predicted to be highly suitable compared to habitats predicted to be not suitable. In other words, ecological niche models help pick out the most frequently occupied habitat of a species. It is not uncommon therefore to expect that it is in these habitats that species would have adapted the most and would be expected to maximize their individual fitness. Our results only confirm this assumption. However, more studies with diverse taxa as case examples may be required to further strengthen and confirm this assumption.

The findings of the study provide a powerful handle for, and direction to, the conservation of rare, endangered and otherwise threatened species. Over the last few years, ecological niche models have become very popular in rationalizing choice of habitat for species re-introduction and ex-situ conservation [77–79]. These choices have rested on the predictions made by the ENMs with respect to the presence/absence of the species in those predicted habitats. However, none of these studies have attempted to test the robustness of the niche predictions with respect to the species fitness in that habitat and the genetic variability of the species. Our results suggest that species re-introductions, species restoration and even domestication of species can be guided by ecological niche models and species thus translocated or domesticated are likely to perform better and have a higher fitness than if they were performed at random. 

Finally with specific reference to *M. malabarica* itself, from our results it is evident that not all regions in the Western Ghats are uniformly suitable for the species. In fact within the Western Ghats, certain areas are highly suitable and others are unsuitable. Areas that have highest “habitat suitability index” would be ideal locations for domestication/cultivation of the species. The Karnataka Forest Department, for example, is keen to enrich the population status of *M. malabarica* in the Western Ghats. The outputs of this study, specifically the prediction of the ENM provide a robust platform for identifying sites for establishing such enrichment plantations.

## Supporting Information

Figure S1 **Distribution records and sampling sites of *M. malabarica* inthe Western Ghats.**
(TIF)

Figure S2 **Density of adults of *M. malabarica* at the sampling sites in the Western Ghats.** Note: Regions represented in black indicate areas of high genetic diversity and allelic richness.
(TIF)

Figure S3 **Map showing genetic diversity parameters of *Myristica malabarica* at sampling locations in the Western Ghats.** A) Allelic richness B) Genetic diversity. Note: Regions represented in black indicate areas of high genetic diversity and allelic richness.
(TIF)

File S1 **Supplementary tables**.
Table **S1**, Geo-coordinates and habitat suitability index of the fourteen populations selected for the study. Table **S2**, Skewnessand Kurtosis tests for frequency distribution of fluctuating asymmetry across highly suitable and poorly suitable habitats.Table **S3**, Kolmogorov-Smirnov (KS) two sample tests for frequency distribution of fluctuating asymmetry and specific leaf weight across highly suitable and poorly suitable habitats. Table **S4**, Skewnessand Kurtosis tests for frequency distribution of specific leaf weight across highly suitable and poorly suitable habitat. Table **S5**, The univariate ANOVA under general linear model (GLM) for different genetic diversity parameters across latitudinal gradient.
(DOC)

## References

[1] Elton C (1927) Animal Ecology. New York: Macmillan Publishers Ltd. p. 256.

[2] Grinnell J (1917) Field tests of theories concerning distributional control. American Naturalist 51: 115–128. doi:10.1086/279591.

[3] Holt RD, Gaines MS (1992) Analysis of adaptation in heterogeneous landscapes: implications for the evolution of fundamental niches. Evolutionary Ecology 6: 433–447. doi:10.1007/BF02270702.

[4] Zizhen L, Hong L (1997) The niche-fitness model of crop population and its application. Ecological Modelling 104: 199–203. doi:10.1016/S0304-3800(97)00127-0.

[5] Maguire BJ (1973) Niche response structure and the analytical potentials of its relationship to the habitat. American Naturalist 107: 213–246. doi:10.1086/282827.

[6] Busby JR (1991) BIOCLIM - a bioclimate analysis and prediction system. Plant Protection Quarterly 6: 8–9.

[7] Stockwell DRB, Noble IR (1992) Induction of sets of rules from animal distribution data: a robust and informative method of data analysis. Mathematics and Computers in Simulation 33: 335–390.

[8] Phillips SJ, Anderson RP, Schapire RE (2006) Maximum entropy modeling of species geographic distributions. Ecological Modelling 190: 231–259. doi:10.1016/j.ecolmodel.2005.03.026.

[9] Peterson AT (2003) Predicting the geography of species’ invasions via ecological niche modeling. Q Rev Biol 78: 419–433. doi:10.1086/378926. PubMed: 14737826. 14737826

[10] Peterson AT, Robins CR (2003) Using ecological-niche modeling to predict Barred Owl invasions with implications for Spotted Owl conservation. Conservation Biology 17: 1161–1165. doi:10.1046/j.1523-1739.2003.02206.x.

[11] Hirzel AH, Le Lay G (2008) Habitat suitability modelling and niche theory. Journal of Applied Ecology 45: 1372–1381. doi:10.1111/j.1365-2664.2008.01524.x.

[12] Pearman PB, Guisan A, Broennimann O, Randin CF (2008) Niche dynamics in space and time. Trends Ecol Evol 23: 149–158. doi:10.1016/j.tree.2007.11.005. PubMed: 18289716. 18289716

[13] Jackson CR, Robertson MP (2011) Predicting the potential distribution of an endangered cryptic subterranean mammal from few occurrence records. Nature Conservation 19: 87–94. doi:10.1016/j.jnc.2010.06.006.

[14] Peterson AT, Martínez-Campos C, Nakazawa Y, Martínez-Meyer E (2005) Time-specific ecological niche modeling predicts spatial dynamics of vector insects and human dengue cases. Trans R Soc Trop Med Hyg 99: 647–655. doi:10.1016/j.trstmh.2005.02.004. PubMed: 15979656. 15979656

[15] Polak T, Saltz D (2011) Reintroduction as an ecosystem restoration technique. Conservation Biology 25: 424 -427. doi:10.1111/j.1523-1739.2011.01669.x. PubMed: 21535145. 21535145

[16] Ganeshaiah KN, Barve N, Nath N, Chandrashekara K, Swamy M et al. (2003) Predicting the potential geographical distribution of the sugarcane woolly aphid using GARP and DIVA-GIS. Current Science 85: 1526–1528.

[17] Barve N, Bonilla AJ, Brandes J, Brown JC, Brunsell N et al. (2012) Climate-change and mass mortality events in overwintering monarch butterflies. Revista Mexicana de Biodiversidad 83: 817–824.

[18] Gurgel-Gonçalves R, Galvão C, Costa J, Peterson AT (2012) Geographic distribution of chagas disease vectors in Brazil based on ecological niche modeling. Journal of Tropical Medicine 2012: 1-15 10.1155/2012/705326 PMC3317230 22523500

[19] Wright JW, Davies KF, Lau JA, McCall AC, McKay JK (2006) Experimental verification of ecological niche modeling in a heterogeneous environment. Ecology 87: 2433–2439. Available online at: doi:10.1890/0012-9658(2006)87[2433:EVOENM]2.0.CO;2. PubMed: 17089652 17089652 10.1890/0012-9658(2006)87[2433:evoenm]2.0.co;2

[20] Martinez-Meyer E, Diaz-Porras D, Peterson AT, Yanez-Arenas C (2012) Ecological niche structure and range wide abundance patterns of species. Biology Letters 9(1): 1-5. 10.1098/rsbl.2012.0637 PMC3565484 23134784

[21] Foden W, Midgley GF, Hughes G, Bond WJ, Thuiller W et al. (2007) A changing climate is eroding the geographical range of the Namib Desert tree Aloe through population declines and dispersal lags. Diversity and Distributions 13: 645–653. doi:10.1111/j.1472-4642.2007.00391.x.

[22] Violle C, Navas M-L, Vile D, Kazakou E, Fortunel C et al. (2007) Let the concept of trait be functional! Oikos 116: 882–892. doi:10.1111/j.0030-1299.2007.15559.x.

[23] Frankham R, Ballou JD, Briscoe DA (2010) Introduction to conservation genetics. 2nd Edition. Cambridge, UK: Cambridge University Press. 644 pp.

[24] Saccheri IJ, Wilson IJ, Nichols RA, Bruford MW, Brakefield PM (1999) Inbreeding of bottlenecked butterfly populations. Estimation using the likelihood of changes in marker allele frequencies. Genetics 151: 1053–1063. PubMed: 10049922. 10049922 10.1093/genetics/151.3.1053 PMC1460528

[25] Bouzat JL, Cheng HH, Lewin HA, Westemeier RL, Brawn JD et al. (1998) Genetic evaluation of a demographic bottleneck in the Greater Prairie Chicken. Conservation Biology 12: 836–843. doi:10.1046/j.1523-1739.1998.97164.x.

[26] Mittermeier RA, Patricio RG, Hoffman M, Pilgrim J, Brooks T et al. (2005) Hotspots revisited: earth’s biologically richest and most endangered terrestrial ecoregions. Conservation International. CEMEX, Mexico City. 392 pp.

[27] Ganeshaiah KN (2003) SasyaSahyadri: distribution, taxonomy and diversity of plants of Western Ghats. UAS, Bangalore, India.

[28] Saldanha CJ (1996) Flora of Karnataka, Vol 2 New Delhi, India: Oxford & IBH Publishing Co. Pvt. Ltd. p. 535.

[29] IUCN (2013) IUCN Red list of threatened species. version 2013.1. Available: www.iucnredlist.org. Accessed 23 September 2013

[30] Krishnamoorthy B, Sasikumar B, Rema J, Johnson K, George P (1997) Genetic resources of tree spices and their conservation in India. Plant Genetic Resources Newsletter 111: 53–58.

[31] Elith J, Graham CH, Anderson RP, Dudík M, Ferrier S et al. (2006) Novel methods improve prediction of species’ distributions from occurrence data. Ecography 29: 129–151. doi:10.1111/j.2006.0906-7590.04596.x.

[32] Saupe EE, Barve V, Myers CE, Soberón J, Barve N et al. (2012) Variation in niche and distribution model performance: the need for a priori assessment of key causal factors. Ecological Modelling 237-238: 11–22. doi:10.1016/j.ecolmodel.2012.04.001.

[33] Hijmans RJ, Cameron SE, Parra JL, Jones PG, Jarvis A (2005) Very high resolution interpolated climate surfaces for global land areas. International Journal of Climatology 25: 1965–1978. doi:10.1002/joc.1276.

[34] Beaumont LJ, Hughes L, Poulsen M (2005) Predicting species distributions: use of climatic parameters in BIOCLIM and its impact on predictions of species’ current and future distributions. Ecological Modelling 186: 251–270. doi:10.1016/j.ecolmodel.2005.01.030.

[35] Barve N, Barve V, Jiménez-Valverde A, Lira-Noriega A, Maher SP et al. (2011) The crucial role of the accessible area in ecological niche modeling and species distribution modeling. Ecological Modelling 222: 1810–1819. doi:10.1016/j.ecolmodel.2011.02.011.

[36] Pearson RG, Raxworthy CJ, Nakamura M, Peterson AT (2006) Predicting species distributions from small numbers of occurrence records: a test case using cryptic geckos in Madagascar. Journal of Biogeography 34: 102–117. doi:10.1111/j.1365-2699.2006.01594.x.

[37] Peterson AT, Papeş M, Soberón J (2008) Rethinking receiver operating characteristic analysis applications in ecological niche modeling. Ecological Modelling 213: 63–72. doi:10.1016/j.ecolmodel.2007.11.008.

[38] Aarssen LW (2008) Death without sex—the “problem of the small” and selection for reproductive economy in flowering plants. Evolutionary Ecology 22: 279–298. doi:10.1007/s10682-007-9170-z.

[39] Christiansen FB, Fenchel TM (1977) Theories of populations in biological communities. New York: Springer-Verlag. 144 pp.

[40] Leimu R, Mutikainen P, Koricheva J, Fischer M (2006) How general are positive relationships between plant population size, fitness and genetic variation? Journal of Ecology 94: 942–952. doi:10.1111/j.1365-2745.2006.01150.x.

[41] Fischer M, Hock M, Paschke M (2003) Low genetic variation reduces cross-compatibility and offspring fitness in populations of a narrow endemic plant with a self-incompatibility system. Conservation Genetics 4: 325–336. doi:10.1023/A:1024051129024.

[42] Markow T, editor (1994) Developmental instability: its origins and evolutionary implications. New York: Springer-Verlag. 441 pp.

[43] Møller AP, Swaddle JP (1998) Asymmetry, developmental stability, and evolution. Oxford University Press, USA. 304 pp.

[44] Møller AP (1992) Female swallow preference for symmetrical male sexual ornaments. Nature 357: 238–240. doi:10.1038/357238a0. PubMed: 1589021. 1589021

[45] Thornhill R (1992) Fluctuating asymmetry and the mating system of the Japanese scorpionfly, *Panorpa* *japonica* . Animal Behaviour 44: 867–879. doi:10.1016/S0003-3472(05)80583-4.

[46] Palmer RA, Strobeck C (1986) Fluctuating asymmetry: measurement, analysis, patterns. Annual Review of Ecology and Systematics 17: 391–421. doi:10.1146/annurev.es.17.110186.002135.

[47] Calvo RN, Horvitz CC (1990) Pollinator limitation, cost of reproduction, and fitness in plants: a transition-matrix demographic approach. American Naturalist 136: 499–516. doi:10.1086/285110.

[48] Fenner M (1992) Seeds: the ecology of regeneration of plant communities. CABI publishing group. 384 p.

[49] Ganeshaiah KN, Uma Shaanker R, Murali KS, Shankar U, Bawa KS (1998) Extraction of non-timber forest products in the forests of BiligiriRangan Hills, India. 5. Influence of dispersal mode on species response to anthropogenic pressures. Economic Botany 52: 316–319. doi:10.1007/BF02862150.

[50] Saccheri I, Kuussaari M, Kankare M, Vikman P, Hanski I (1998) Inbreeding and extinction in a butterfly metapopulation. Nature 392: 491–494. doi:10.1038/33136.

[51] Wisely SM, Buskirk SW, Fleming MA, McDonald DB, Ostrander EA (2002) Genetic diversity and fitness in Black-footed Ferrets before and during a bottleneck. J Hered 93: 231–237. doi:10.1093/jhered/93.4.231. PubMed: 12407208. 12407208

[52] Slate J, Kruuk LE, Marshall TC, Pemberton JM, Clutton-Brock TH (2000) Inbreeding depression influences lifetime breeding success in a wild population of red deer (*Cervuselaphus*). Proceedings of the Royal Society of London B 267: 1657–1662. doi:10.1098/rspb.2000.1192. PMC1690715 11467429

[53] Doyle JJ, Doyle JL (1987) A rapid DNA isolation procedure for small quantities of fresh leaf tissue. Phytochemical Bulletin 19: 11–15.

[54] Hemmilä S, Kumara M, Ravikanth G, Gustafsoon S, Vasudeva R et al. (2010) Development of eleven microsatellite markers in the red-listed tree species *Myristica* *malabarica* . Conservation Genetics Resources 2: 305–307. doi:10.1007/s12686-010-9212-7.

[55] El Mousadik A, Petit RJ (1996) High level of genetic differentiation for allelic richness among populations of the argan tree [*Arganiaspinosa* (L.) Skeels] endemic to Morocco. Theoretical and Applied Genetics 92: 832–839. doi:10.1007/BF00221895. PubMed: 24166548. 24166548

[56] Goudet J (1995) Fstat: a computer program to calculate F-Statistics. Journal of Heredity 86: 485–486.

[57] Kalinowski ST (2005) HP-RARE 1.0: a computer program for performing rarefaction on measures of allelic richness. Molecular Ecology Notes 5: 187–189.

[58] Raymond M, Rousset F (1995) GENEPOP (Version1.2): population genetics software for exact tests and ecumenicism. Journal of Heredity 86: 248–249.

[59] Sokal RR, Rohlf FJ (1994) Biometry: the principles and practices of statistics in biological research. 3rd Ed.. W. H. Freeman. 880 pp.

[60] Christensen R (1996) Plane answers to complex questions. 3rd ed. Springer-Verlag.452 pp.

[61] Adhikari D, Barik SK, Upadhaya K (2012) Habitat distribution modelling for reintroduction of *Ilex* *khasiana*Purk., a critically endangered tree species of northeastern India. Ecological Engineering 40: 37–43. doi:10.1016/j.ecoleng.2011.12.004.

[62] Elmendorf SC, Moore KA (2008) Use of community-composition data to predict the fecundity and abundance of species. Conserv Biol 22: 1523–1532. doi:10.1111/j.1523-1739.2008.01051.x. PubMed: 18847440. 18847440

[63] Thuiller W, Albert CH, Dubuis A, Randin C, Guisan A (2010) Variation in habitat suitability does not always relate to variation in species’ plant functional traits. Biol Lett 6: 120–123. doi:10.1098/rsbl.2009.0669. PubMed: 19793738. 19793738 PMC2817270

[64] Fischer M, Matthies D (1997) Mating structure and inbreeding and outbreeding depression in the rare plant *Genetianellagermanica* (Gentianaceae). Am J Bot 84: 1685–1692. doi:10.2307/2446466. PubMed: 21708572. 21708572

[65] Dostálek T, Münzbergová Z, Plačková I (2010) Genetic diversity and its effect on fitness in an endangered plant species, *Dracocephalumaustriacum* L. Conservation Genetics 11: 773–783. doi:10.1007/s10592-009-9879-z.

[66] Hensen I, Oberprieler C, Wesche K (2005) Genetic structure, population size, and seed production of *Pulsatilla* *vulgaris* Mill. (Ranunculaceae) in Central Germany. Flora - Morphology, Distribution, Functional Ecology of Plants 200: 3–14. doi:10.1016/j.flora.2004.05.001.

[67] Wilsey BJ, Haukioja E, Koricheva J, Sulkinoja M (1998) Leaf fluctuating asymmetry increases with hybridization and elevation in tree-line birches. Ecology 79: 2092–2099. Available online at: doi:10.1890/0012-9658(1998)079[2092:LFAIWH]2.0.CO;2

[68] Valkama J, Kozlov MV (2001) Impact of climatic factors on the developmental stability of mountain birch growing in a contaminated area. Journal of Applied Ecology 38: 665–673. doi:10.1046/j.1365-2664.2001.00628.x.

[69] Nagamitsu T, Kawahara T, Hotta M (2004) Phenotypic variation and leaf fluctuating asymmetry in isolated populations of an endangered dwarf birch *Betulaovalifolia* in Hokkaido, Japan. Plant Species Biology 19: 13–21. doi:10.1111/j.1442-1984.2004.00097.x.

[70] Kozlov MV, Wilsey BJ, Koricheva J, Haukioja E (1996) Fluctuating asymmetry of birch leaves increases under pollution impact. Journal of Applied Ecology 33: 1489–1495. doi:10.2307/2404787.

[71] Jurik T, Chabot BF, Chabot JF (1977) Effects of changing the light regime during leaf development on photosynthetic performance of *Fragariavirginiania* . Bulletin of the Ecological Society of America 58: 32.

[72] Steinbauer MJ, Clarke AR, Madden JL (1998) Oviposition preference of a Eucalyptus herbivore and the importance of leaf age on interspecific host choice. Ecological Entomology 23: 201–206. doi:10.1046/j.1365-2311.1998.00122.x.

[73] Howlett BG, Clarke AR, Madden JL (2001) The influence of leaf age on the oviposition preference of *Chrsophthartabimaculata* (Olivier) and the establishment of neonates. Agricultural and Forest Entomology 3: 121–127. doi:10.1046/j.1461-9563.2001.00096.x.

[74] Lewontin RC (1974) The genetic basis of evolutionary change. New York: Columbia University Press. 352 pp.

[75] Dixon AL, Herlihy CR, Busch JW (2013) Demographic and population-genetic tests provide mixed support for the abundant centre hypothesis in the endemic plant *Leavenworthiastylosa* . Mol Ecol 22: 1777–1791. doi:10.1111/mec.12207. PubMed: 23356549. 23356549

[76] Albert CH, Thuiller W (2008) Favourability functions versus probability of presence: advantages and misuses. Ecography 31: 417–422. doi:10.1111/j.0906-7590.2008.05221.x.

[77] Danks FS, Klein DR (2002) Using GIS to predict potential wildlife habitat: a case study of muskoxen in northern Alaska. International Journal of Remote Sensing 23: 4611–4632. doi:10.1080/01431160110113890.

[78] Carroll C, Phillips MK, Schumaker NH, Smith DW (2003) Impacts of landscape change on wolf restoration success: planning a reintroduction program based on static and dynamic spatial models. Conservation Biology 17: 536–548. doi:10.1046/j.1523-1739.2003.01552.x.

[79] Armstrong DP, Ewen JG (2002) Dynamics and viability of a New Zealand Robin population reintroduced to regenerating fragmented habitat. Conservation Biology 16: 1074–1085. doi:10.1046/j.1523-1739.2002.00215.x.

