# Supplementary figures and images for: Do Ecological Niche Model Predictions Reflect the Adaptive Landscape of Species?: A Test Using Myristica malabarica Lam., an Endemic Tree in the Western Ghats, India
Source: PLoS One. 2013 Nov 29;8(11):e82066. doi: 10.1371/journal.pone.0082066 (PMC3843714; doi:10.1371/journal.pone.0082066)

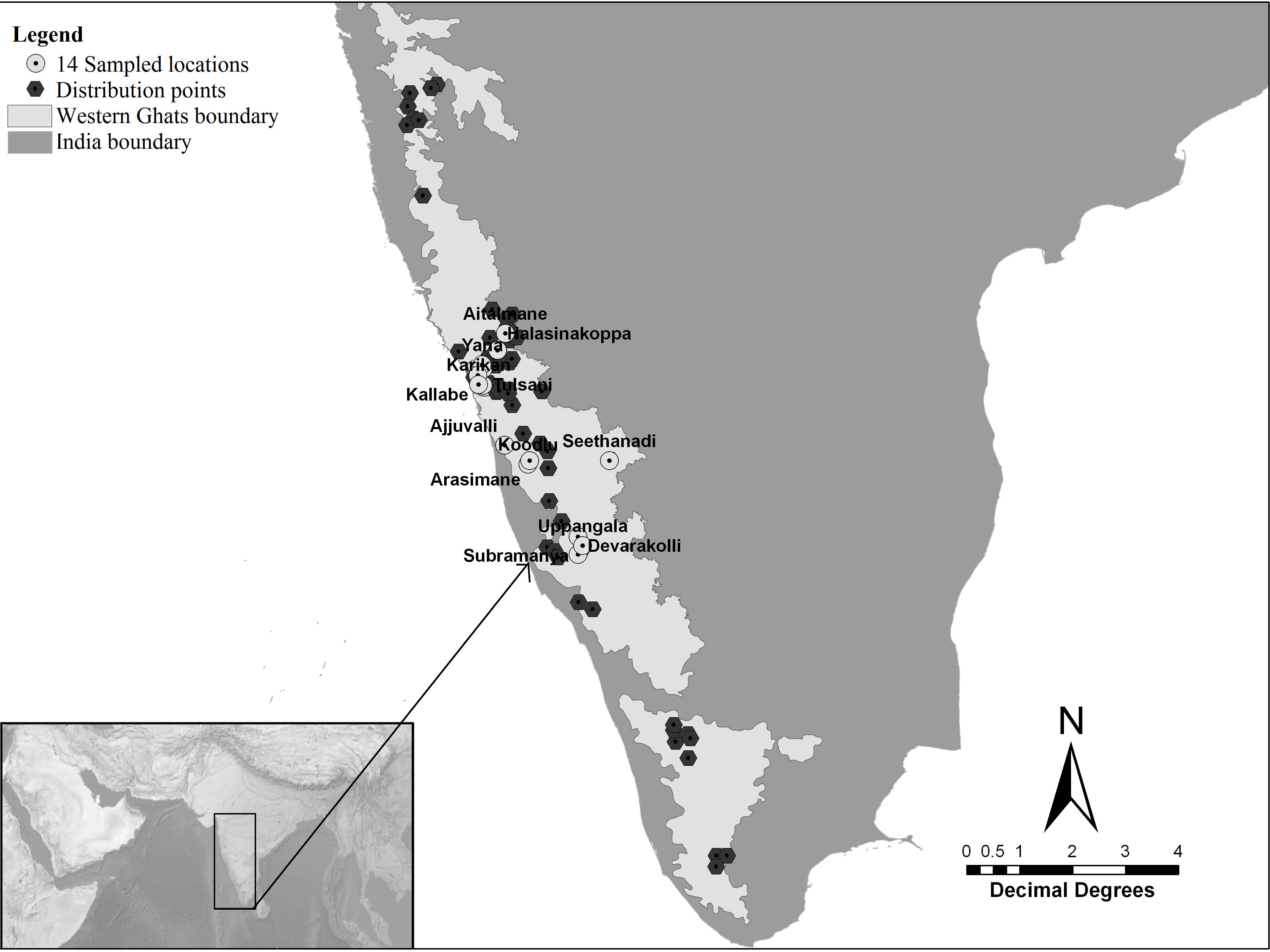

Supplement: Figure S1 — Distribution records and sampling sites of M. malabarica inthe Western Ghats. (TIF) [file pone.0082066.s001.tif]

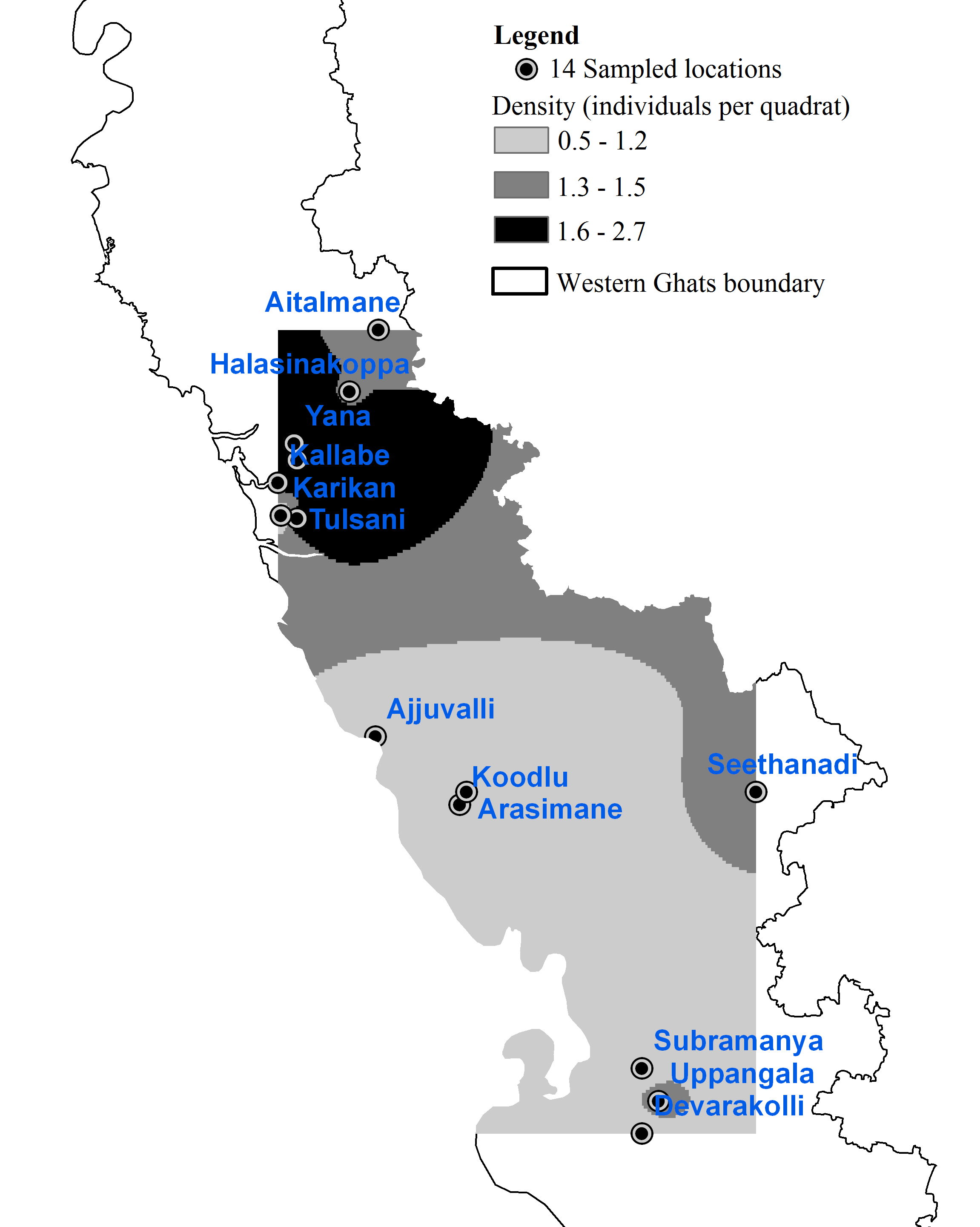

Supplement: Figure S2 — Density of adults of M. malabarica at the sampling sites in the Western Ghats. Note: Regions represented in black indicate areas of high genetic diversity and allelic richness. (TIF) [file pone.0082066.s002.tif]

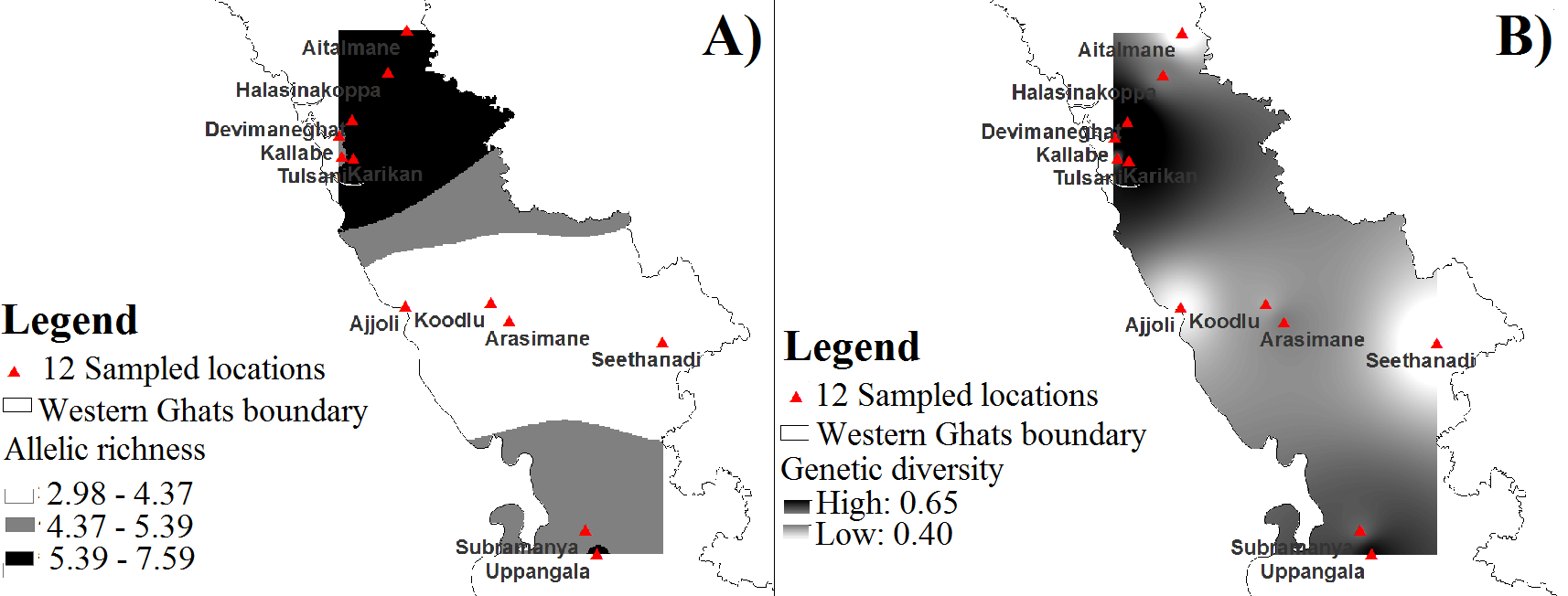

Supplement: Figure S3 — Map showing genetic diversity parameters of Myristica malabarica at sampling locations in the Western Ghats. A) Allelic richness B) Genetic diversity. Note: Regions represented in black indicate areas of high genetic diversity and allelic richness. (TIF) [file pone.0082066.s003.tif]
