# Supplementary material for: Do Ecological Niche Model Predictions Reflect the Adaptive Landscape of Species?: A Test Using Myristica malabarica Lam., an Endemic Tree in the Western Ghats, India
Source: PLoS One. 2013 Nov 29;8(11):e82066. doi: 10.1371/journal.pone.0082066 (PMC3843714; doi:10.1371/journal.pone.0082066)
Supplement: File S1 — Supplementary tables. Table S1, Geo-coordinates and habitat suitability index of the fourteen populations selected for the study. Table S2, Skewnessand Kurtosis tests for frequency distribution of fluctuating asymmetry across highly suitable and poorly suitable habitats.Table S3, Kolmogorov-Smirnov (KS) two sample tests for frequency distribution of fluctuating asymmetry and specific leaf weight across highly suitable and poorly suitable habitats. Table S4, Skewnessand Kurtosis tests for frequency distribution of specific leaf weight across highly suitable and poorly suitable habitat. Table S5, The univariate ANOVA under general linear model (GLM) for different genetic diversity parameters across latitudinal gradient. (DOC) [file pone.0082066.s004.doc]

**Table S1:** Geo-coordinates and habitat suitability index of the fourteen populations selected for the study.

|  |  |  | **Habitat suitability indices** | |
| --- | --- | --- | --- | --- |
| **Population** | **Latitude (^0^N)** | **Longitude (^0^E)** | **Bioclim** | **Maxent** |
| Halasinakoppa | 14.73 | 74.71 | 221 | 83 |
| Aitalmane | 14.92 | 74.8 | 100 | 71 |
| Devimane Ghat | 14.52 | 74.55 | 164 | 75 |
| Yana* | 14.57 | 74.54 | 43 | 68 |
| Tulsani | 14.34 | 74.55 | 114 | 72 |
| Devarakolli* | 12.45 | 75.61 | 86 | 60 |
| Koodlu | 13.46 | 75.05 | 86 | 57 |
| Arasimane | 13.5 | 75.07 | 86 | 54 |
| Kallabe | 14.45 | 74.49 | 43 | 57 |
| Karikan | 14.35 | 74.5 | 114 | 87 |
| Seethanadi | 13.5 | 75.96 | *21* | *6* |
| Ajjuvalli | 13.67 | 74.79 | *21* | *43* |
| Subramanya | 12.65 | 75.61 | *50* | *30* |
| Uppangala | 12.55 | 75.66 | 164 | 60 |

*****Populations not included for genetic analysis.

**Table S2:** Skewness and Kurtosis tests for frequency distribution of fluctuating asymmetry across highly suitable and poorly suitable habitats.

|  | **Bioclim** | | | | **Maxent** | | | |
| --- | --- | --- | --- | --- | --- | --- | --- | --- |
|  | **Highly suitable habitat** | | **Poorly suitable habitat** | | **Highly suitable habitat** | | **Poorly suitable habitat** | |
| Permutation number | Skewness/kurtosis | p-value | Skewness/Kurtosis | p-value | Skewness/Kurtosis | p-value | Skewness/Kurtosis | p-value |
| 1 | 1.953/5.37 | **<0.0001** | 2.016/7.23 | **<0.0001** | 1.83/4.62 | **<0.0001** | 1.50/3.74 | **<0.0001** |
| 2 | 2.653/10.6 | **<0.0001** | 1.91/7.45 | **<0.0001** | 1.80/4.17 | **<0.0001** | 2.74/13.0 | **<0.0001** |
| 3 | 2.95/13.13 | **<0.0001** | 2.26/9.16 | **<0.0001** | 1.80/4.07 | **<0.0001** | 2.27/8.62 | **<0.0001** |
| 4 | 1.75/3.56 | **<0.0001** | 1.44/3.31 | **<0.0001** | 1.88/5.07 | **<0.0001** | 2.03/9.23 | **<0.0001** |
| 5 | 2.37/9.55 | **<0.0001** | 2.61/12.66 | **<0.0001** | 1.92/5.06 | **<0.0001** | 2.35/11.17 | **<0.0001** |
| 6 | 2.48/9.36 | **<0.0001** | 1.39/3.07 | **<0.0001** | 1.77/3.98 | **<0.0001** | 2.04/7.43 | **<0.0001** |
| 7 | 1.79/4.14 | **<0.0001** | 2.08/7.89 | **<0.0001** | 2.04/5.30 | **<0.0001** | 2.40/9.12 | **<0.0001** |
| 8 | 3.00/13.80 | **<0.0001** | 1.83/6.96 | **<0.0001** | 1.30/0.97 | **<0.0001** | 1.10/1.59 | **<0.0001** |
| 9 | 2.53/10.19 | **<0.0001** | 2.37/8.87 | **<0.0001** | 1.72/5.87 | **<0.0001** | 0.85/0.95 | **<0.0001** |
| 10 | 2.37/8.81 | **<0.0001** | 0.94/0.77 | **<0.0001** | 1.76/3.23 | **<0.0001** | 2.59/9.93 | **<0.0001** |
| 11 | 2.78/13.48 | **<0.0001** | 1.96/8.33 | **<0.0001** | 1.92/4.68 | **<0.0001** | 2.64/12.48 | **<0.0001** |
| 12 | 2.22/6.86 | **<0.0001** | 1.92/6.40 | **<0.0001** | 1.78/4.47 | **<0.0001** | 1.59/3.93 | **<0.0001** |
| 13 | 2.76/12.21 | **<0.0001** | 1.98/6.85 | **<0.0001** | 1.52/2.65 | **<0.0001** | 2.47/10.12 | **<0.0001** |
| 14 | 2.04/5.51 | **<0.0001** | 1.90/7.74 | **<0.0001** | 1.98/5.04 | **<0.0001** | 1.51/4.55 | **<0.0001** |
| 15 | 2.55/9.49 | **<0.0001** | 2.27/7.86 | **<0.0001** | 1.91/4.79 | **<0.0001** | 2.20/10.57 | **<0.0001** |
| 16 | 2.23/9.04 | **<0.0001** | 0.72/-0.177 | **<0.0001** | 1.75/4.09 | **<0.0001** | 2.12/7.72 | **<0.0001** |
| 17 | 2.55/6.44 | **<0.0001** | 1.31/2.83 | **<0.0001** | 1.79/5.10 | **<0.0001** | 0.76/0.003 | **<0.0001** |
| 18 | 2.42/10.05 | **<0.0001** | 2.27/8.81 | **<0.0001** | 1.79/3.75 | **<0.0001** | 2.37/8.49 | **<0.0001** |
| 19 | 2.45/8.82 | **<0.0001** | 2.16/8.35 | **<0.0001** | 1.50/3.19 | **<0.0001** | 2.30/8.80 | **<0.0001** |
| 20 | 2.54/10.89 | **<0.0001** | 1.72/6.02 | **<0.0001** | 2.06/5.20 | **<0.0001** | 2.04/9.23 | **<0.0001** |
| 21 | 2.42/9.10 | **<0.0001** | 1.65/5.69 | **<0.0001** | 1.99/4.57 | **<0.0001** | 2.28/10.50 | **<0.0001** |
| 22 | 2.51/10.42 | **<0.0001** | 2.31/9.41 | **<0.0001** | 1.12/0.76 | **<0.0001** | 2.09/7.83 | **<0.0001** |
| 23 | 2.83/12.96 | **<0.0001** | 2.19/8.56 | **<0.0001** | 1.80/4.55 | **<0.0001** | 2.04/7.99 | **<0.0001** |
| 24 | 2.03/5.39 | **<0.0001** | 1.48/3.75 | **<0.0001** | 1.89/4.59 | **<0.0001** | 2.34/10.15 | **<0.0001** |
| 25 | 2.41/8.71 | **<0.0001** | 0.90/0.42 | **<0.0001** | 1.89/4.57 | **<0.0001** | 2.62/11.97 | **<0.0001** |

**Table S3:** Kolmogorov-Smirnov (KS) two sample tests for frequency distribution of fluctuating asymmetry and specific leaf weight across highly suitable and poorly suitable habitats.

|  | **Fluctuating Asymmetry** | | | | **Specific Leaf Weight** | | | |
| --- | --- | --- | --- | --- | --- | --- | --- | --- |
| Permutation number | Bioclim | | Maxent | | Bioclim | | Maxent | |
|  | D-value | p-value | D-value | p-value | D-value | p-value | D-value | p-value |
| 1 | 0.128 | **0.026** | 0.262 | **<0.0001** | 0.281 | **<0.0001** | 0.535 | **<0.0001** |
| 2 | 0.111 | 0.077 | 0.218 | **0.0003** | 0.255 | **<0.0001** | 0.571 | **<0.0001** |
| 3 | 0.08 | 0.68 | 0.241 | **<0.0001** | 0.279 | **<0.0001** | 0.571 | **<0.0001** |
| 4 | 0.177 | **0.0005** | 0.217 | **0.0003** | 0.272 | **<0.0001** | 0.535 | **<0.0001** |
| 5 | 0.093 | 0.301 | 0.260 | **<0.0001** | 0.306 | **<0.0001** | 0.584 | **<0.0001** |
| 6 | 0.142 | **0.009** | 0.220 | **0.0003** | 0.244 | **<0.0001** | 0.522 | **<0.0001** |
| 7 | 0.116 | **0.055** | 0.232 | **0.0002** | 0.245 | **<0.0001** | 0.502 | **<0.0001** |
| 8 | 0.122 | **0.038** | 0.242 | **<0.0001** | 0.309 | **<0.0001** | 0.604 | **<0.0001** |
| 9 | 0.105 | 0.109 | 0.245 | **<0.0001** | 0.302 | **<0.0001** | 0.556 | **<0.0001** |
| 10 | 0.130 | **0.022** | 0.225 | **0.0001** | 0.235 | **<0.0001** | 0.549 | **<0.0001** |
| 11 | 0.116 | **0.055** | 0.273 | **<0.0001** | 0.269 | **<0.0001** | 0.579 | **<0.0001** |
| 12 | 0.122 | **0.037** | 0.201 | **0.0012** | 0.268 | **<0.0001** | 0.529 | **<0.0001** |
| 13 | 0.142 | **0.009** | 0.256 | **<0.0001** | 0.269 | **<0.0001** | 0.488 | **<0.0001** |
| 14 | 0.093 | 0.204 | 0.202 | **0.0011** | 0.278 | **<0.0001** | 0.618 | **<0.0001** |
| 15 | 0.143 | **0.008** | 0.212 | **0.0005** | 0.257 | **<0.0001** | 0.569 | **<0.0001** |
| 16 | 0.103 | 0.121 | 0.245 | **<0.0001** | 0.300 | **<0.0001** | 0.545 | **<0.0001** |
| 17 | 0.108 | 0.088 | 0.188 | **0.0032** | 0.248 | **<0.0001** | 0.562 | **<0.0001** |
| 18 | 0.126 | **0.029** | 0.290 | **<0.0001** | 0.295 | **<0.0001** | 0.544 | **<0.0001** |
| 19 | 0.178 | **0.0004** | 0.172 | **0.0094** | 0312 | **<0.0001** | 0.532 | **<0.0001** |
| 20 | 0.06 | 0721 | 0.267 | **<0.0001** | 0.248 | **<0.0001** | 0.574 | **<0.0001** |
| 21 | 0.140 | **0.011** | 0.228 | **0.0001** | 0.240 | **<0.0001** | 0.515 | **<0.0001** |
| 22 | 0.099 | 0.147 | 0.229 | **0.0001** | 0.303 | **<0.0001** | 0.590 | **<0.0001** |
| 23 | 0.147 | **0.006** | 0.229 | **0.0001** | 0.297 | **<0.0001** | 0.586 | **<0.0001** |
| 24 | 0.091 | 0.217 | 0.225 | **0.0001** | 0.252 | **<0.0001** | 0.520 | **<0.0001** |
| 25 | 0.155 | **0.003** | 0.227 | **0.0001** | 0.292 | **<0.0001** | 0.535 | **<0.0001** |

**Table S4:** Skewness and Kurtosis tests for frequency distribution of specific leaf weight across highly suitable and poorly suitable habitat.

|  | **BIOCLIM** | | | | **MAXENT** | | | |
| --- | --- | --- | --- | --- | --- | --- | --- | --- |
| Permutation number | High | | Poor | | High | | Poor | |
|  | Skewness/kurtosis | p-value | Skewness/Kurtosis | p-value | Skewness/Kurtosis | p-value | Skewness/Kurtosis | p-value |
| 1 | -0.291/0.633 | **0.041/0.026** | 0.650/1.540 | **<0.0001** | 0.528/0.977 | **0.0006/0.001** | **0.037/-0.588** | 0.849/0.136 |
| 2 | -0.615/0.496 | **<0.0001/**0.08 | 0.497/0.795 | **0.0004/0.0048** | 0.449/0.855 | **0.004/0.006** | 0.070/-0.141 | 0.718 |
| 3 | -0.464/0.464 | **0.001/**0.103 | 0.656/1.240 | **<0.0001** | 0.315/0.764 | **0.042/0.013** | -0.113/-0.287 | 0.568/0.465 |
| 4 | -0436/0.709 | **0.002/**0.013 | 0.401/0.735 | **0.0046/0.009** | 0.538/0.817 | **0.0006/0.0082** | 0.163/-0.526 | 0.412/0.183 |
| 5 | -0.423/0.561 | **0.003/0.048** | 0.554/1.041 | **<0.0001/0.00** | 0.609/1.320 | **<0.0001** | 0.151/-0.763 | 0.447/0.053 |
| 6 | -0.465/0.577 | **0.0012/0.042** | 0.583/1.233 | **<0.0001** | 0.419/0.690 | **0.007/0.026** | -0.023/0.129 | **0.904/0.741** |
| 7 | -0.487/0.160 | **0.0006/**0.568 | 0.654/1.462 | **<0.0001** | 0.442/0.545 | **0.004/**0.078 | 0.102/-0.0019 | **0.603/0.992** |
| 8 | -0.369/1.029 | **0.009/0.0004** | 0.467/0.705 | **0.001/0.012** | 0.587/1.437 | **0.0002/<0.00** | 0.019/-0.744 | 0.920/0.060 |
| 9 | -0.480/0.534 | **0.0008/**0.060 | 0.675/1.537 | **<0.0001** | 0.690/1378 | **<0.0001** | 0.129/-0.218 | 0.515/0.575 |
| 10 | -0.416/0.612 | **0.003/0.031** | 0.457/0.846 | **0.0012/0.002** | 0.308/0.548 | **0.046**/0.076 | -0.024/-0.595 | 0.896/-0.595 |
| 11 | -0.406/0.629 | **0.004/0.027** | 0.374/0.546 | **0.008/0.052** | 0.561/0.925 | **0.0004/0.002** | 0.131/0.035 | 0.509/0.928 |
| 12 | -0.483/0.513 | **0.0008/**0.070 | 0.819/1.922 | **<0.0001** | 0.449/1.031 | **0.004/0.0008** | 0.001/-0.755 | 0.922/0.056 |
| 13 | -0.797/0.511 | **<0.0001/**0.07 | 0.736/1.636 | **<0.0001** | 0.671/1.498 | **<0.0001** | 0.001/-0.249 | 0.992/0.528 |
| 14 | -0.180/0.532 | **0.207/**0.061 | 0.356/0.393 | **0.011/0.161** | 0.308/0.321 | **0.046/0.298** | 0.0921/-0.540 | 0.645/0.170 |
| 15 | -0.305/0.432 | **0.032/**0.128 | 0.576/1.188 | **<0.0001** | 0.432/0.793 | **0.005/0.010** | -0.030/-0.496 | 0.873/0.212 |
| 16 | -0.604/0.763 | **<0.0001/0.00** | 0.573/1.070 | **<0.0001** | 0.581/1.184 | **0.0002/0.000** | 0.122/-0.328 | 0.535/0.406 |
| 17 | -0.279/0.654 | **0.050/0.021** | 0.443/1.126 | **0.002/<0.000** | 0.207/-0.178 | **0.298/0.653** | 0.457/1.229 | 0.003/<0.0 |
| 18 | -0.615/0.504 | **<0.0001/**0.07 | 0.690/1.175 | **<0.0001** | 0.536/0.761 | **0.0006/0.013** | -0.077/-0.582 | 0.697/0.14 |
| 19 | -0.587/1.129 | **<0.0001** | 0.330/0.532 | **0.019/0.058** | 0.626/1.361 | **<0.0001** | 0.143/-0.836 | 0.471/0.03 |
| 20 | -0.330/0.157 | **0.020/**0.575 | 0747/1.452 | **<0.0001** | 0.382/0.653 | **0.014/0.034** | -0.039/0.250 | 0.841/0.52 |
| 21 | -0.298/0.510 | **0.036/**0.073 | 0.592/1.247 | **<0.0001** | 0.466/0.859 | **0.0025/0.005** | -0.010/-0.229 | 0.952/0.56 |
| 22 | -0.597/0.686 | **<0.0001/0.01** | 0.554/1.051 | **<0.0001** | 0.546/1.111 | **0.0004/0.000** | 0.128/-0.527 | 0.522/0.18 |
| 23 | -0.378/0.452 | **0.008/0.111** | 0.774/1.354 | **<0.0001** | 0.520/0.815 | **0.0008/0.008** | 0.029/-0.400 | 0.880/0.31 |
| 24 | -0.517/0.704 | **0.0002/0.013** | 0.326/0.878 | **0.020/0.0018** | 0.482/1.159 | **0.002/0.0002** | 0.049/-0.408 | 0.802/0.30 |
| 25 | -0.599/0.676 | **<0.0001/0.01** | 0.592/0.746 | **<0.0001/0.00** | 0.459/0.748 | **0.003/0**.0157 | -0.020/-0.145 | 0.912/0.69 |

**Table S5**: The univariate ANOVA under general linear model (GLM) for different genetic diversity parameters across latitudinal gradient.

| **Dependent variable** | **F(df)** | ***r*** | **p** |
| --- | --- | --- | --- |
| Gene diversity per locus | 0.465(11) | 0.089 | 0.498 |
| Observed number of alleles (NA) | 7.483(11) | 0.338 | 0.008 |
| Allelic richness (AR) | 2.437(11) | 0.201 | 0.124 |
| Number of private alleles (AP) | 2.827(11) | 0.215 | 0.098 |
| Inbreeding co-efficient (Fis) | 1.213 (11) | 0.143 | 0.245 |
| Pair-wise Fst | 27.55(11) | -0.418 | <0.0001 |
